# Editorial: Connecting the Dots Between Obesity, Diabetes and Cancer

**DOI:** 10.3389/fendo.2020.583456

**Published:** 2020-11-19

**Authors:** Xuemei Tong, Mei Kong, Debbie C. Thurmond

**Affiliations:** ^1^ Department of Biochemistry and Molecular Cell Biology, Shanghai Key Laboratory for Tumor Microenvironment and Inflammation, Key Laboratory of Cell Differentiation and Apoptosis of Chinese Ministry of Education, Shanghai Jiao Tong University School of Medicine, Shanghai, China; ^2^ Department of Molecular Biology and Biochemistry, School of Biological Sciences, University of California, Irvine, CA, United States; ^3^ Department of Molecular and Cellular Endocrinology, Diabetes and Metabolism Research Institute, Beckman Research Institute of City of Hope, Duarte, CA, United States

**Keywords:** diabetes, obesity, cancer, metabolic disease, metabolism

n the past several decades, the prevalence of obesity, diabetes, and cancer has become one of the greatest health burdens worldwide (1, 2). Obesity, diabetes, and cancer are metabolic diseases with detrimental health consequences. Moreover, epidemiological studies suggest that people with diabetes and obesity are at significantly higher risk for many types of cancer including liver, colorectal, breast, and pancreatic cancer (3, 4). Therefore, clinicians often face the situation of treating patients with cancer and diabetes or obesity. In addition, many drugs used in the treatment of diabetes have different effects on obesity and cancer. For example, metformin treatment has been reported to reduce the risk of cancer and obesity while insulin has been associated with an increased risk of cancer (5). Conversely, treatment of cancer using checkpoint inhibitors (e.g., PD-L1 inhibitors) is associated with increased risk of insulin-dependent diabetes (6, 7). Therefore, the complex relationship between obesity, diabetes and cancer has been studied intensively. In this issue, we have assembled a series of up-to-date reviews and original research on the connections between obesity, diabetes, and cancer.

Diet induces alterations in systemic and cellular metabolic profiles. Western diet high in fats and fructose is one of the main causes for the prevalence of many metabolic diseases (8, 9). In addition to the well-established relationship between high fat diet and the metabolic syndrome, recent studies have shed light on how fructose influences obesity, diabetes and cancer (10). Strober and Brady reviewed recent findings on the role of increased dietary fructose consumption in shifting the metabolic state of the whole body and in specific tissues, which had profound effects on many diseases, including obesity, diabetes, and cancer.

Hyperinsulinemia or insulin resistance is common in diabetic or obese patients (11). After binding to the insulin receptor present in most of mammalian cell types, insulin activates cell signaling pathways such as the PI3K/AKT/mTOR and the RAS/RAF/mitogen activated protein kinase (MAPK) pathway (12). Therefore, in addition to being a key regulator of metabolism, insulin plays an important role in cellular homeostasis. Yee et al. summarized the central role of the insulin signaling pathway in diabetes and cancer by regulating glycolysis, ROS levels, inflammation, cell migration, and angiogenesis. The review has also pointed out the importance of treating insulin resistance for oncologists because the insulin signaling is critical for promoting cancer initiation and progression.

Decreased skeletal muscle mass with reduced glucose uptake and catabolism contributes to whole body metabolic dysfunction in obesity, diabetes, and cancer (13, 14). Therefore, increased skeletal muscle growth with improved metabolic efficiency and exercise performance will benefit systemic metabolism and overall health of patients with metabolic diseases. Berdeaux and Hutchins discussed the challenge of specifically targeting the CREB (cAMP response element binding protein) and CRTCs (CREB regulated transcriptional coactivators) in the skeletal muscle without disturbing CREB-CRTCs in other organs or cancer cells. One solution is to identify skeletal muscle specific regulatory mechanisms controlling CREB-CRTC transcriptional activity to safely improve health in patients with obesity, diabetes, and cancer.

The number and function of mitochondria is largely controlled by GTPases such as Mfn1, Mfn2, Opa1, and Drp1 which regulate the fusion and fission of the organelle (15). Since mitochondrial abnormalities are usually observed in patients with obesity, diabetes and cancer, Mfn1, Mfn2, Opa1, and Drp1 are tightly associated with the development of these diseases (16, 17). Dai and Jiang updated the metabolic roles of these GTPases in obesity, diabetes, and cancer by balancing fusion and fission of mitochondria and orchestrating mitochondrial number and function.

Among many dysregulated metabolic pathways in obesity, diabetes and cancer, the pentose phosphate pathway (PPP) is drawing more and more attention. Being the major source for NADPH and *de novo* nucleotide biosynthesis, the PPP is critical for regulating the redox homeostasis and macromolecular biosynthesis (18, 19). Ge et al. pointed out that PPP enzymes including G6PD, 6PGD, and TKT could serve as promising targets for prevention and treatment of metabolic diseases. 

In order to explore the role of obesity-induced mechanical pressure in lipid metabolism and insulin sensitivity, Zhao et al. studied the function of Piezo1, an adipocyte-rich cation channel stimulated by membrane tension and stretch, in adipose tissues. Adipose-specific Piezo1 knockout mice were prone to diet-induced metabolic diseases including obesity, insulin resistance and hepatic steatosis by triggering pro-inflammatory responses.

Our series of reviews and original research summarized some recent progress in elucidating metabolic, hormonal and immune connections between obesity, diabetes and cancer. Given the epidemic of the diseases, reducing cancer occurrence in obese or diabetic patients, and in addition, reducing the prevalence of metabolic syndrome that associates with increased risk for cancer, will be crucial for greatly improving global health.

## Author Contributions

XT, MK, and DT wrote the editorial. All authors contributed to the article and approved the submitted version.

## Conflict of Interest

The authors declare that the research was conducted in the absence of any commercial or financial relationships that could be construed as a potential conflict of interest.
